# Supplementary material for: Seminoma and Embryonal Carcinoma Footprints Identified by Analysis of Integrated Genome-Wide Epigenetic and Expression Profiles of Germ Cell Cancer Cell Lines
Source: PLoS One. 2014 Jun 2;9(6):e98330. doi: 10.1371/journal.pone.0098330 (PMC4041891; doi:10.1371/journal.pone.0098330)
Supplement: File S1 — ZIP file containing DMRforPairs output for significant regions. Please start from the html files. (ZIP) [file pone.0098330.s008.zip › figures/1997.pdf]

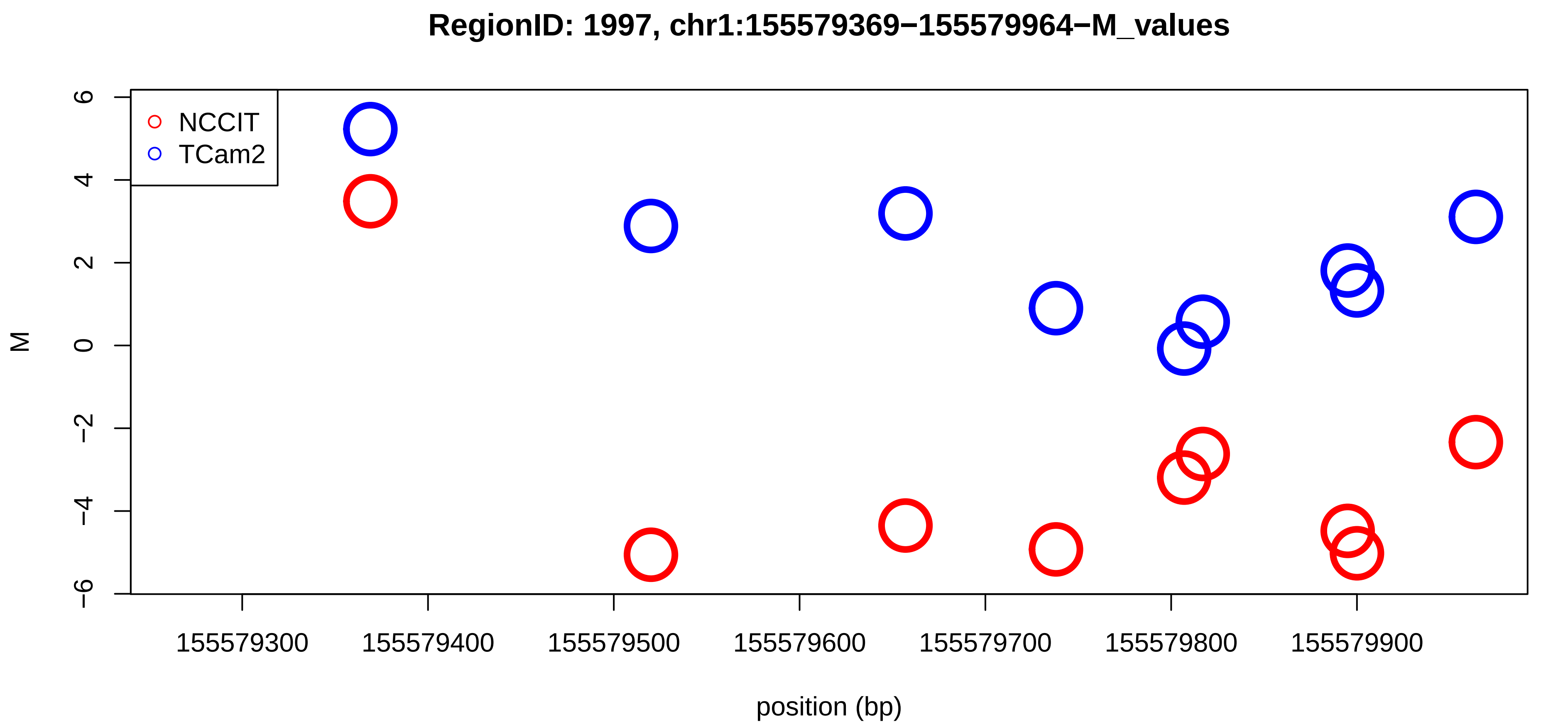

RegionID: 1997, chr1:155579369−155579964−M_values
NCCIT
TCam2
M
6
4
2
0
−2
−4
−6
155579300
155579400
155579500
155579600
155579700
155579800
155579900
position (bp)


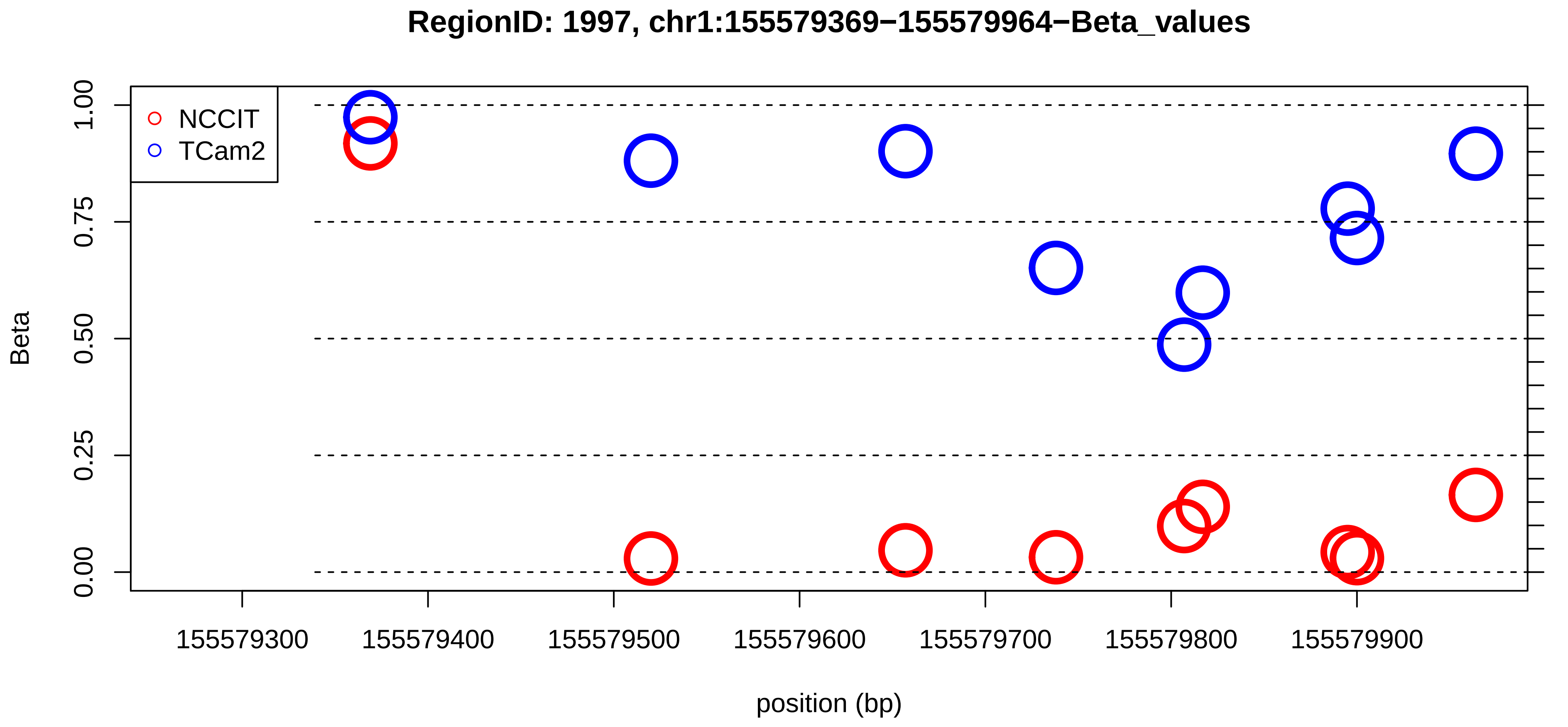

RegionID: 1997, chr1:155579369−155579964−Beta_values
NCCIT
TCam2
Beta
1.00
0.75
0.50
0.25
0.00
155579300
155579400
155579500
155579600
155579700
155579800
155579900
position (bp)
